# Synthesis, Structural Characterization, and Hydrogen Release of Al-Based Amidoboranes Derived from MAlH_4_ (Li, Na)-BH_3_NH_2_CH_2_CH_2_NH_2_BH_3_

**DOI:** 10.3390/molecules30071559

**Published:** 2025-03-31

**Authors:** Ting Zhang, Xiao Li, Hai-Wen Li, Michel Devillers, Yaroslav Filinchuk

**Affiliations:** 1 Institute of Condensed Matter and Nanosciences, Université Catholique de Louvain, Place Louis Pasteur 1, 1348 Louvain-la-Neuve, Belgium; tingzhang9101@foxmail.com (T.Z.); lixiao_hgmri@163.com (X.L.);; 2 Hefei General Machinery Research Institute, Hefei 230031, China; 3 School of Advanced Energy, Sun Yat-Sen University, Shenzhen 518107, China; lihw76@mail.sysu.edu.cn

**Keywords:** Al-based amidoboranes, multi-metallic amidoboranes, thermal dehydrogenation, hydrogen release

## Abstract

Over the past two decades, the high hydrogen content and favorable dehydrogenation conditions of multi-metallic amidoboranes have gained significant attention for their potential in hydrogen storage. Among them, Al-based complex hydrides have shown promise because of their high polarizing power, light weight, and abundant natural presence. In this work, we successfully synthesized two novel tetrahedrally coordinated Al-based amidoboranes, namely, Li[Al(BH_3_NHCH_2_CH_2_NHBH_3_)_2_] and Na(THF)[Al(BH_3_NHCH_2_CH_2_NHBH_3_)_2_], using BH_3_NH_2_CH_2_CH_2_NH_2_BH_3_ (EDAB) as a precursor. The structure of Na(THF)[Al(BH_3_NHCH_2_CH_2_NHBH_3_)_2_] was determined through modeling based on synchrotron powder X-ray diffraction. Additionally, the formation of the Al-N bond in Li[Al(BH_3_NHCH_2_CH_2_NHBH_3_)_2_] and Na(THF)[Al(BH_3_NHCH_2_CH_2_NHBH_3_)_2_] was confirmed with IR spectra. Na(THF)[Al(BH_3_NHCH_2_CH_2_NHBH_3_)_2_] is more stable in air than Li[Al(BH_3_NHCH_2_CH_2_NHBH_3_)_2_]. Importantly, thermal gravimetric analysis and mass spectroscopic characterization confirmed that both compounds release hydrogen without the presence of ammonia, diborane, or ethylenediamine. Our work represents the first example of Al-based amidoboranes with chelation coordination geometry, which provides an essential foundation for understanding the relationship of complex multi-metallic amidoboranes in terms of synthesis, structure, and properties.

## 1. Introduction

As global energy consumption continues to rise toward its peak, fossil fuel resources are being depleted worldwide, resulting in escalating energy costs and increased greenhouse gas emissions [1]. Hydrogen has emerged as a promising alternative energy source, characterized by its high energy density (120 MJ/kg), sustainability, and environmentally friendly profile. It can be produced [2,3] from both renewable and non-renewable sources and is widely applicable in transportation, power generation, and industrial sectors. Despite these advantages, the commercialization of hydrogen energy is significantly constrained by its low volumetric density under standard conditions [4], which complicates storage and transportation. Conventional approaches, such as high-pressure gas storage and cryogenic liquid storage [5], partially address this issue but are associated with high energy consumption, safety risks, and system complexity. In recent years, solid-state hydrogen storage [6,7,8,9,10] has gained considerable attention as a viable alternative, offering advantages such as high storage capacity, enhanced safety, stability, and operation under moderate temperature and pressure conditions, thereby presenting a potential solution to the limitations of traditional storage methods.

Compounds based on boron–nitrogen–hydrogen (B-N-H) [11,12] have emerged as promising solid-state hydrogen storage materials due to the lightweight nature of boron and nitrogen, as well as their ability to accommodate multiple hydrogen atoms. This is attributed to the hydridic and protic characteristics of the B-H and N-H bonds, which facilitate the release of hydrogen. One representative example of B-N-H material is ammonia borane [13,14] (NH_3_BH_3_ or AB), which can release up to 19.6 wt.% of hydrogen, exhibits low toxicity, and demonstrates good stability under ambient conditions.

However, the thermal decomposition of NH_3_BH_3_ occurs in multiple steps, typically at around 120 °C, 200 °C, and above 500 °C, leading to the generation of toxic volatile by-products such as B_2_H_6_, NH_3_, and N_3_B_3_H_6_. The decomposition process is often accompanied by severe foaming and volume expansion. Various strategies [15,16] have been explored to address these limitations, including the chemical modifications of NH_3_BH_3_ molecules to create derivatives. This approach has resulted in the synthesis of novel compounds like alkali amidoboranes [17,18,19,20], alkaline earth amidoboranes [21,22,23,24,25], and multi-metallic amidoboranes [26,27,28,29,30,31,32,33,34,35,36,37], which exhibit improved characteristics for hydrogen storage applications. For instance, KNH_2_BH_3_ releases approximately 5.8 wt.% of pure hydrogen from room temperature up to 160 °C [18]. Mg(NH_2_BH_3_)_2_ can release approximately 10 wt.% of high-purity H_2_ upon heating to 300 °C [25]. Furthermore, Na_2_Mg(NH_2_BH_3_)_4_ has the ability to release 8.4 wt.% of predominantly hydrogen, with minor amounts of ammonia and borazine [28].

Compared to monometallic amidoboranes, multi-metallic amidoboranes offer greater versatility in forming new compounds. So far, multi-metallic amidoboranes have been categorized into Li-, Mg-, Ca-, and Al-based amidoboranes according to the central coordination metals. Our works focus on the synthesis and chemical modification of Al-based amidoboranes, driven by the high polarizing power defined by the exceptional charge-to-radius ratio, low weight, and high natural abundance of Al.

Our initial investigation on Al-based amidoboranes led to the discovery of Na[Al(NH_2_BH_3_)_4_] [34], which exhibits a two-step hydrogen release process (at 120 °C and 168 °C), releasing approximately 9 wt.% of high-purity hydrogen. Furthermore, the amorphous residue obtained after the thermal decomposition of Na[Al(NH_2_BH_3_)_4_] demonstrated the ability to reversibly absorb around 27% of the released hydrogen at 250 °C and a hydrogen pressure of 150 bar. Subsequently, we conducted a study on the formation, structure, and hydrogen release properties of its derivative, Na[Al(CH_3_NHBH_3_)_4_] [37]. This compound was synthesized from NaAlH_4_ and CH_3_NH_2_BH_3_ (MeAB), and the introduction of a methyl group (-CH_3_) on the nitrogen of NH_3_BH_3_ stabilized an intermediate compound, Na[AlH(CH_3_NHBH_3_)_3_], shedding light on the formation process of amidoboranes. Notably, the energy input required for the formation of Na[Al(CH_3_NHBH_3_)_4_] was lower than that of the unsubstituted Na[Al(NH_2_BH_3_)_4_]. Na[Al(CH_3_NHBH_3_)_4_] does not release high-purity hydrogen during its thermal decomposition but forms reactive hydride composites with NaH and NaNH_2_, desorbing pure H_2_ under relatively mild conditions.

To further advance the development of Al-based amidoboranes and gain a deeper understanding of the relationships between synthesis, structure, and hydrogen release properties, we conducted additional modifications to Na[Al(NH_2_BH_3_)_4_]. In this paper, we introduce the new Al-based amidoboranes, namely, Li[Al(BH_3_NHCH_2_CH_2_NHBH_3_)_2_] and Na(THF)[Al(BH_3_NHCH_2_CH_2_NHBH_3_)_2_], providing comprehensive details about their synthesis, structural characterization, and thermal dehydrogenation properties.

For the synthesis of Li[Al(BH_3_NHCH_2_CH_2_NHBH_3_)_2_] and Na(THF)[Al(BH_3_NHCH_2_CH_2_NHBH_3_)_2_], we employed BH_3_NH_2_CH_2_CH_2_NH_2_BH_3_ (EDAB) as one of the precursors. EDAB offers greater accessibility than NH_3_BH_3_, as it can be obtained from commercially available and cost-effective precursors. Additionally, the presence of the weak electron-donating ethylene substituent influences the coordination bonds between the nitrogen and aluminum atoms in the final complex, similar to the behavior observed in Na[Al(CH_3_NHBH_3_)_4_]. EDAB exhibits a dimer-like structure derived from CH_3_NH_2_BH_3_, facilitating the formation of a chelate complex with aluminum. This characteristic contributes to the creation of a more stable framework for Al-based amidoboranes, suppressing the release of significant fragments during thermal decomposition, as observed in Na[Al(CH_3_NHBH_3_)_4_] [37].

## 2. Results

### 2.1. Synthesis of M[Al(BH_3_NHCH_2_CH_2_NHBH_3_)_2_] (M = Li and Na)

#### 2.1.1. Synthesis of Li[Al(BH_3_NHCH_2_CH_2_NHBH_3_)_2_]

The crystal structures of the reported Al-based amidoborane complexes in a tetrahedral coordination geometry include Na[Al(NH_2_BH_3_)_4_] [34], K[Al(NH_2_BH_3_)_4_] [36], Na[AlH(CH_3_NHBH_3_)_3_] [37], and Na[Al(CH_3_NHBH_3_)_4_] [37]. These complexes can be synthesized by ball-milling NaAlH_4_ or KAlH_4_ with four equivalents of NH_3_BH_3_ or CH_3_NH_2_BH_3_. Mechanochemical synthesis is preferred over wet chemical methods for the synthesis of hydrides used for hydrogen storage materials, as it avoids the use of coordinating or high-boiling-point solvents, which can increase the costs and the environmental impact.

It has been reported that the reaction between one equivalent of LiAlH_4_ and four equivalents of NH_3_BH_3_ under ball milling conditions is excessively violent [34]. Furthermore, the lower melting point of CH_3_NH_2_BH_3_ (58 °C) [38] compared to NH_3_BH_3_ (112–114 °C) [39] results in NaAlH_4_-4 CH_3_NH_2_BH_3_ composites, exhibiting higher reactivity than NaAlH_4_-4 NH_3_BH_3_. As a result, ball milling may not be suitable for the synthesis of Li[Al(CH_3_NHBH_3_)_4_]. In contrast, BH_3_NH_2_CH_2_CH_2_NH_2_BH_3_ (EDAB) demonstrates higher thermal stability and a slightly higher melting point (119 °C) [40] compared to NH_3_BH_3_ and CH_3_NH_2_BH_3_. Therefore, the reaction between LiAlH_4_ and EDAB is expected to go easier than between LiAlH_4_ and CH_3_NH_2_BH_3_ and/or NH_3_BH_3_. Thus, we attempted the ball-milling synthesis of a novel Al-based amidoborane complex with Li using LiAlH_4_ and EDAB.

**Figure 1:**
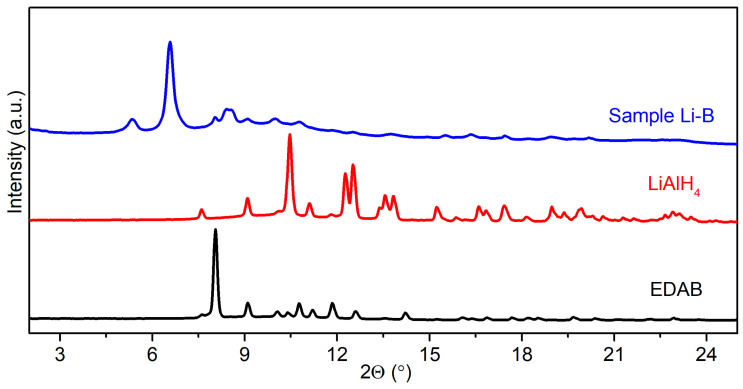
PXRD patterns of EDAB, LiAlH_4_, and Sample Li-B (λ = 0.71073 Å).

Initially, we conducted ball milling experiments using LiAlH_4_-2 EDAB composites under the same conditions as for the synthesis of Na[Al(NH_2_BH_3_)_4_]; however, the analysis of the powder X-ray diffraction (PXRD) pattern revealed that the reaction between LiAlH_4_ and 2 EDAB could not reach completion under these milling conditions (details shown in Section 3.2, Sample **Li-A**). To enhance the yield of the new product from the post-milled LiAlH_4_-2 EDAB composites, we optimized the milling conditions (details shown in Section 3.2, Sample **Li-B**) and obtained a higher yield for Li[Al(BH_3_NHCH_2_CH_2_NHBH_3_)_2_] (Figure 1, Sample **Li-B**).

**Figure 2:**
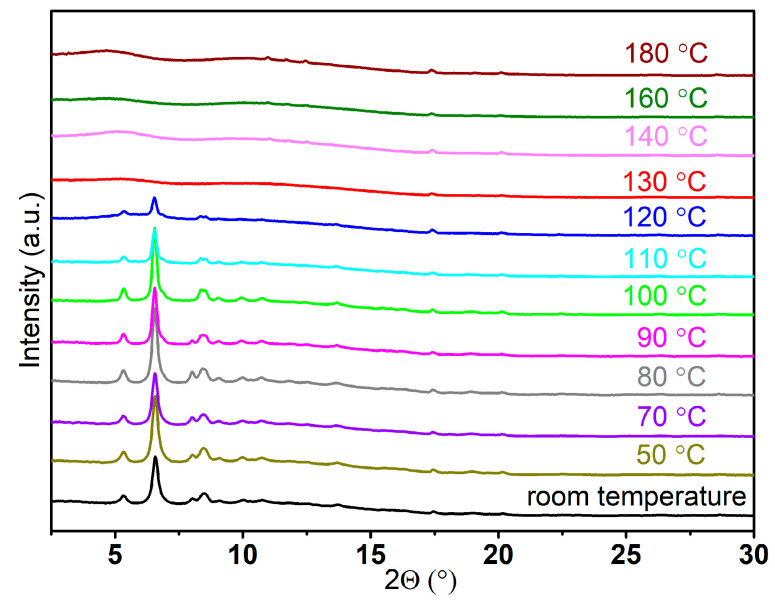
Temperature ramp followed by PXRD for Sample Li-B (λ = 0.71073 Å).

Variable-temperature PXRD measurements were conducted to determine the number of phases associated with the newly observed peaks in Sample **Li-B** (Figure 1). Interestingly, all the peaks observed in the PXRD pattern of Sample **Li-B** (Figure 1) vanished simultaneously at approximately 130 °C (Figure 2). This indicates that these peaks correspond to a single phase. The pressure and temperature were carefully monitored throughout the milling process of LiAlH_4_-2 EDAB (refer to Section 3.2 and Table 1). The results revealed the release of approximately 3.3 equivalents of gas per mole of LiAlH_4_, which closely matches the expected gas quantity (four equivalents) based on the equation below.

LiAlH_4_ + 2BH_3_NH_2_CH_2_CH_2_NH_2_BH_3_→Li[Al(BH_3_NHCH_2_CH_2_NHBH_3_)_2_] + 4 H_2_↑

We also performed an IR spectra analysis of the Li[Al(BH_3_NHCH_2_CH_2_NHBH_3_)_2_] (Sample **Li-B**), comparing it with that of Na(THF)[Al(BH_3_NHCH_2_CH_2_NHBH_3_)_2_]. Interestingly, the IR spectra of Li[Al(BH_3_NHCH_2_CH_2_NHBH_3_)_2_] exhibited a striking similarity to that of Na(THF)[Al(BH_3_NHCH_2_CH_2_NHBH_3_)_2_]. This observation, combined with the findings from ramping PXRD measurements and the analysis of released gases during synthesis, strongly suggests that Li[Al(BH_3_NHCH_2_CH_2_NHBH_3_)_2_] was successfully synthesized under the milling conditions employed for Sample **Li-B**.

#### 2.1.2. Synthesis of Na(THF)[Al(BH_3_NHCH_2_CH_2_NHBH_3_)_2_]

In our quest to obtain Al-based amidoboranes with Na^+^ as a counterion, we explored the wet chemical synthesis approach by reacting NaAlH_4_ and EDAB. Previous reports indicated that Na[Al(NH_2_BH_3_)_4_] could be obtained in THF at room temperature within 3 h [41]. Following this, we conducted the synthesis of Na(THF)[Al(BH_3_NHCH_2_CH_2_NHBH_3_)_2_] in anhydrous THF with varying stirring times. Specifically, we combined one eq. of NaAlH_4_ and two eq. of EDAB in anhydrous THF at room temperature and allowed the reaction to proceed for 24, 48, and 72 h, yielding, respectively, Samples **Na-A**, **Na-B**, and **Na-C** (details are provided about the synthesis method in Section 3.3).

**Figure 3:**
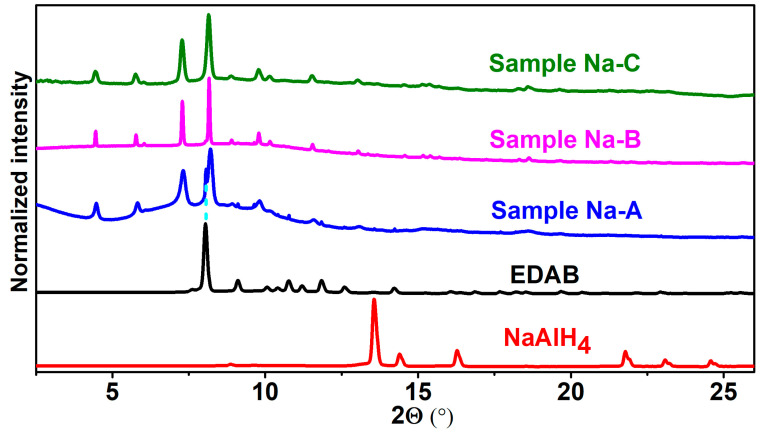
PXRD patterns of NaAlH_4_, EDAB, and Samples Na-A, B, and C (the residual EDAB in Sample Na-A is represented by a light blue dashed line, λ = 0.71073 Å).

Our results demonstrate that Na(THF)[Al(BH_3_NHCH_2_CH_2_NHBH_3_)_2_] could indeed be formed in THF at room temperature, albeit with a longer reaction time compared to its analog Na[Al(NH_2_BH_3_)_4_]. We monitored the progress of the reaction using PXRD patterns (Figure 3). After 24 h of reaction (Sample **Na-A**), new peaks appeared on the PXRD, indicating the formation of new compounds. Extending the reaction time to 48 and 72 h (Samples **Na-B** and **Na-C**), the peaks attributed to EDAB completely disappeared, while the new peaks matched those observed in Sample **Na-A** (Figure 3). This confirms that the reaction between NaAlH_4_ and EDAB was completed within 48 h.

**Figure 4:**
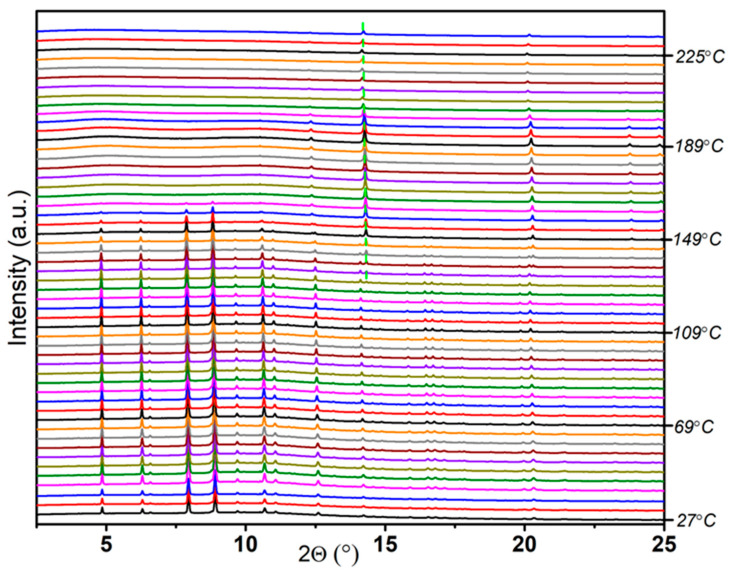
Temperature ramping PXRD pattern of Na(THF)[Al(BH_3_NHCH_2_CH_2_NHBH_3_)_2_] (the green dotted line represents the peaks of NaBH_4_, λ = 0.77509 Å).

To verify the purity of the newly synthesized compound, we performed temperature ramping synchrotron PXRD from room temperature to 225 °C (Figure 4). The results demonstrated that all the peaks observed for Sample **Na-B** gradually decreased simultaneously from approximately 121 °C and eventually vanished at 165 °C. This observation suggests that Sample **Na-B** consists of a single phase, confirming its purity.

We also attempted to synthesize Na(THF)[Al(BH_3_NHCH_2_CH_2_NHBH_3_)_2_] via mechanochemistry. Unfortunately, all the attempts were unsuccessful, despite employing the harshest milling conditions in our ball mill. Considering the significantly longer stirring time of Na(THF)[Al(BH_3_NHCH_2_CH_2_NHBH_3_)_2_] required for synthesis (over 24 h) compared to the synthesis of Na[Al(NH_2_BH_3_)_4_] and the unsuccessful mechanochemical synthesis, both indicate that EDAB exhibits higher inertness than AB when reacting with NaAlH_4_. It was reported that MeAB has a higher reactivity than AB with respect to NaAlH_4_ [37]. Consequently, MeAB exhibits the highest activity, and EDAB exhibits the highest inertness among AB, MeAB, and EDAB in the reaction with NaAlH_4_.

### 2.2. Characterization of M[Al(BH_3_NHCH_2_CH_2_NHBH_3_)_2_] (M = Li, Na)

For structural characterization, we employed synchrotron PXRD data and conducted modeling to determine the structure of this novel Al-based amidoborane complex, Na(THF)[Al(BH_3_NHCH_2_CH_2_NHBH_3_)_2_]. Additionally, we analyzed the IR spectra to provide additional support for our structural conclusions.

#### 2.2.1. The Structure of Na(THF)[Al(BH_3_NHCH_2_CH_2_NHBH_3_)_2_]

The crystal structure of Li[Al(BH_3_NHCH_2_CH_2_NHBH_3_)_2_] could not be determined due to the limited number of diffraction peaks and their significant broadening. Therefore, the structure of Li[Al(BH_3_NHCH_2_CH_2_NHBH_3_)_2_] was characterized through IR spectra, which will be discussed later.

Fortunately, the crystal structure of Na(THF)[Al(BH_3_NHCH_2_CH_2_NHBH_3_)_2_] with THF was successfully determined using synchrotron powder X-ray diffraction (SR-PXRD, ESRF, Grenoble, France). Although the disorder of the coordinated THF remains poorly resolved, the structural framework has been well determined. Based on the SR-PXRD data, the crystal structure of Na(THF)[Al(BH_3_NHCH_2_CH_2_NHBH_3_)_2_] with THF was modeled in a tetragonal unit cell with space group P*4/n*. In this anionic complex, each [BH_3_NHCH_2_CH_2_NHBH_3_]^2−^ anion provides two pairs of electrons from two nitrogen atoms, forming a chelate coordinated with the central Al^3+^ ions. This arrangement contributes to the enhanced stability of the compound Na(THF)[Al(BH_3_NHCH_2_CH_2_NHBH_3_)_2_] compared to its analogs, Na[Al(NH_2_BH_3_)_4_] and Na[Al(CH_3_NHBH_3_)_4_].

**Figure 5:**
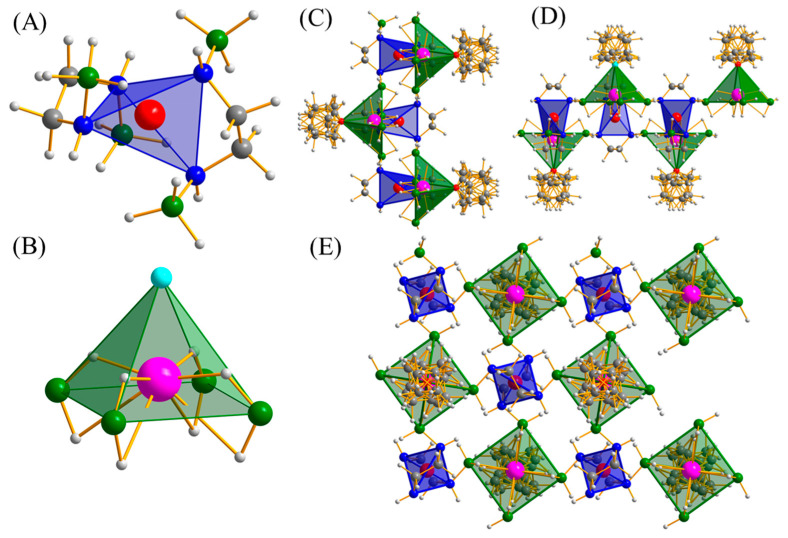
The coordinated geometry of Al^3+^ (**A**) and Na^+^ (**B**) crystal packing along with a (**C**), b (**D**), and c (**E**) axes (color code: N = blue, B = green, C = grey, H = light grey, O = sky blue, Al = red, and Na = pink).

The central Al^3+^ ion is tetrahedrally coordinated by four nitrogen atoms (Figure 5A), exhibiting a geometry similar to previously reported compounds such as Na[Al(NH_2_BH_3_)_4_] [34], K[Al(NH_2_BH_3_)_4_] [36], and Na[Al(CH_3_NHBH_3_)_4_] [37]. The Al-N distances were fixed at 1.95 Å, slightly longer than those observed in Na[Al(NH_2_BH_3_)_4_] (1.840(9)–1.929(8) Å) and K[Al(NH_2_BH_3_)_4_] (1.838(9)–1.909(9) Å) but similar to those in Na[Al(CH_3_NHBH_3_)_4_] (1.922(1)–1.990(1) Å).

The Na atoms exhibit a square pyramidal coordination geometry (Figure 5B), with each atom surrounded by four BH_3_ groups from four different [Al(BH_3_NHCH_2_CH_2_NHBH_3_)_2_]^−^ anions, as well as one oxygen atom from the THF solvent molecule. This arrangement differs from the usual octahedral environments observed in compounds like Na[Al(NH_2_BH_3_)_4_] [34], Na_2_[Mg(NH_2_BH_3_)_4_] [28], and Na_2_[Ca(NH_2_BH_3_)_4_] [31], as well as the triangular bipyramidal environment in Na[Al(CH_3_NHBH_3_)_4_] [37].

The THF exhibits disorder around the four-fold symmetry axis, which could be better resolved by studying a single crystal. Similar to the reported Na[Al(NH_2_BH_3_)_4_] and Na[Al(CH_3_NHBH_3_)_4_], the [BH_3_NHCH_2_CH_2_NHBH_3_]^2−^ units display a bridging coordination mode, connecting Al^3+^ and Na^+^ ions, resulting in the formation of a three-dimensional polymeric structure (Figure 5C–E).

#### 2.2.2. The IR Spectra of M[Al(BH_3_NHCH_2_CH_2_NHBH_3_)_2_] (M = Li and Na)

**Figure 6:**
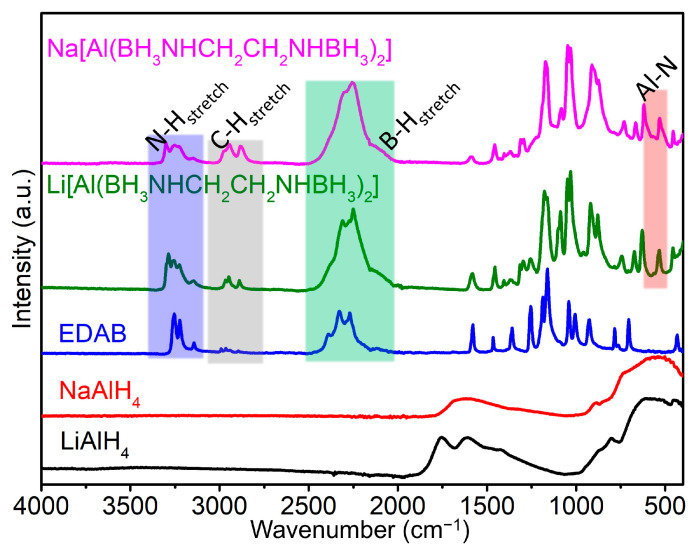
The IR spectra of LiAlH_4_, NaAlH_4_, EDAB, Li[Al(BH_3_NHCH_2_CH_2_NHBH_3_)_2_], and Na(THF)[Al(BH_3_NHCH_2_CH_2_NHBH_3_)_2_] (the stretch bond areas of N-H, C-H, and B-H are represented by blue, gray, and green rectangles; the Al-N bond is represented by a red rectangle).

To characterize the structure of Li[Al(BH_3_NHCH_2_CH_2_NHBH_3_)_2_] and compare it with Na(THF)[Al(BH_3_NHCH_2_CH_2_NHBH_3_)_2_], we conducted ATR-IR spectra measurements on LiAlH_4_, NaAlH_4_, EDAB, Li[Al(BH_3_NHCH_2_CH_2_NHBH_3_)_2_], and Na(THF)[Al(BH_3_NHCH_2_CH_2_NHBH_3_)_2_]. The IR spectra of Li[Al(BH_3_NHCH_2_CH_2_NHBH_3_)_2_] and Na(THF)[Al(BH_3_NHCH_2_CH_2_NHBH_3_)_2_] exhibit characteristic peaks in comparison to the reactant EDAB, indicating structural differences between these compounds (Figure 6).

The IR spectra show the presence of stretching modes for N-H (3114–3346 cm^−1^), C-H (2838–3010 cm^−1^), and B-H (2033–2499 cm^−1^) in both Li[Al(BH_3_NHCH_2_CH_2_NHBH_3_)_2_] and Na(THF)[Al(BH_3_NHCH_2_CH_2_NHBH_3_)_2_]. There is no significant difference compared to the precursor EDAB. Additionally, new peaks appear in these two new complexes within the Al-N bond region (highlighted by the red rectangle) between 400 and 800 cm^−1^, similar to other compounds such as Li[Al(NH_2_)_4_] [42], Na[Al(NH_2_)_4_] [43,44], Na[Al(NH_2_BH_3_)_4_] [34], and Na[Al(CH_3_NHBH_3_)_4_] [37]. All of the above suggest that Li[Al(BH_3_NHCH_2_CH_2_NHBH_3_)_2_] has a similar tetrahedrally coordinated Al-N-chelated geometry.

Furthermore, the intensity of C-H stretching in Na(THF)[Al(BH_3_NHCH_2_CH_2_NHBH_3_)_2_] is higher than that in Li[Al(BH_3_NHCH_2_CH_2_NHBH_3_)_2_] and EDAB (as indicated by the gray area in Figure 6). This observation can be attributed to the presence of the C-H bonds of the THF molecules coordinating with Na^+^ in Na(THF)[Al(BH_3_NHCH_2_CH_2_NHBH_3_)_2_]. This finding is consistent with the structural analysis and the results of thermal decomposition. The latter will be discussed in the following section.

### 2.3. Thermal Dehydrogenation of M[Al(BH_3_NHCH_2_CH_2_NHBH_3_)_2_] (M = Li and Na)

#### 2.3.1. Thermal Dehydrogenation of Li[Al(BH_3_NHCH_2_CH_2_NHBH_3_)_2_]

**Figure 7:**
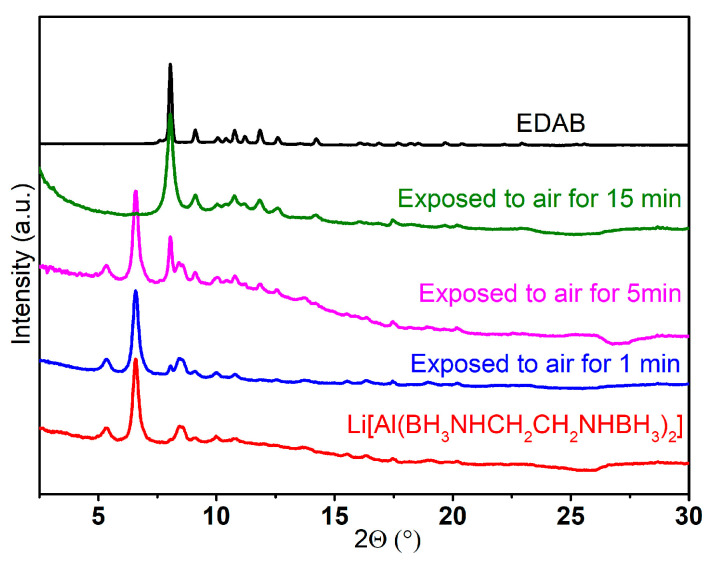
PXRD pattern of EDAB, Li[Al(BH_3_NHCH_2_CH_2_NHBH_3_)_2_], and Li[Al(BH_3_NHCH_2_CH_2_NHBH_3_)_2_] exposed to air for 1, 5, and 15 min (λ = 0.71073 Å).

Before studying the thermal decomposition of Li[Al(BH_3_NHCH_2_CH_2_NHBH_3_)_2_], we conducted a PXRD analysis to assess its stability in the air. The results revealed that Li[Al(BH_3_NHCH_2_CH_2_NHBH_3_)_2_] is highly sensitive to air. After being exposed to air for just a minute, the peaks attributed to the free EDAB noticeably increased. Furthermore, after 15 min, the signals corresponding to Li[Al(BH_3_NHCH_2_CH_2_NHBH_3_)_2_] completely disappeared (Figure 7). Only the peaks of EDAB were observed in the PXRD pattern, indicating that the Al-N bond was broken upon the contact of Li[Al(BH_3_NHCH_2_CH_2_NHBH_3_)_2_] with air.

**Figure 8:**
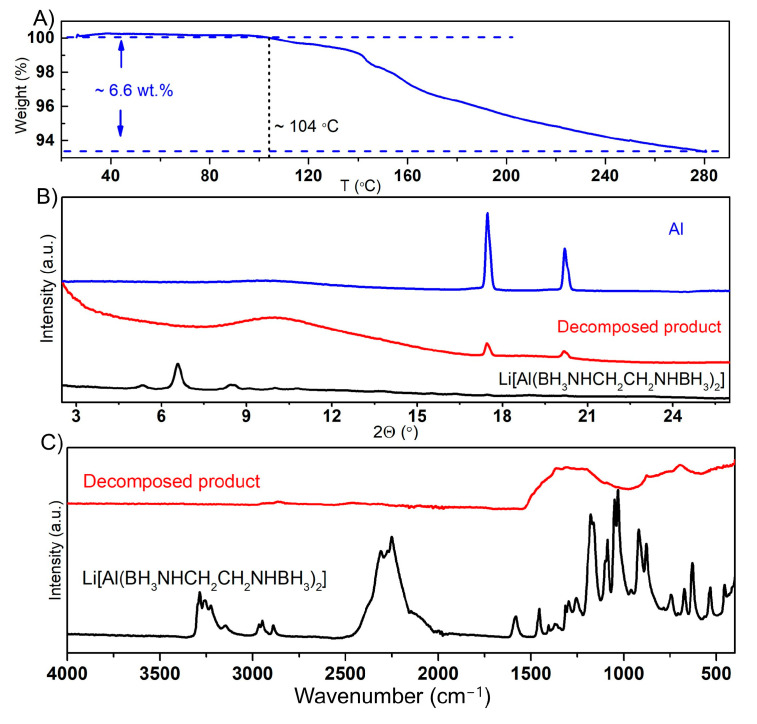
Thermogravimetric analysis (TGA) of Li[Al(BH_3_NHCH_2_CH_2_NHBH_3_)_2_] (**A**); PXRD pattern of Li[Al(BH_3_NHCH_2_CH_2_NHBH_3_)_2_], the thermally decomposed product, and aluminum (**B**); ATR-IR spectra of Li[Al(BH_3_NHCH_2_CH_2_NHBH_3_)_2_] and of the thermally decomposed product (**C**) (λ = 0.71073 Å).

Due to the extreme instability of Li[Al(BH_3_NHCH_2_CH_2_NHBH_3_)_2_] in air, its thermal stability was examined using thermogravimetric analysis (TGA) under an inert argon atmosphere, covering a temperature range from room temperature to 280 °C. The TGA results showed that the thermal decomposition of Li[Al(BH_3_NHCH_2_CH_2_NHBH_3_)_2_] started at approximately 104 °C. In contrast to the starting compound EDAB [40], the thermal decomposition of Li[Al(BH_3_NHCH_2_CH_2_NHBH_3_)_2_] did not exhibit weight oscillation (jet effect), as can be seen in Figure 8A, which is considered a drawback of the thermal dehydrogenation of NH_3_BH_3_ and its derivatives.

Based on PXRD and IR analyses, the solid decomposition products obtained after heating at 280 °C were identified as amorphous phase(s) lacking N-H and B-H bonds but containing metallic aluminum (Figure 8B,C). The mass loss during the thermal decomposition of Li[Al(BH_3_NHCH_2_CH_2_NHBH_3_)_2_] was approximately 6.6 wt.%, which is within the expected value for the release of pure H_2_ (7.8% for theoretical hydrogen content of Li[Al(BH_3_NHCH_2_CH_2_NHBH_3_)_2_], excluding hydrogen on carbon).

**Figure 9:**
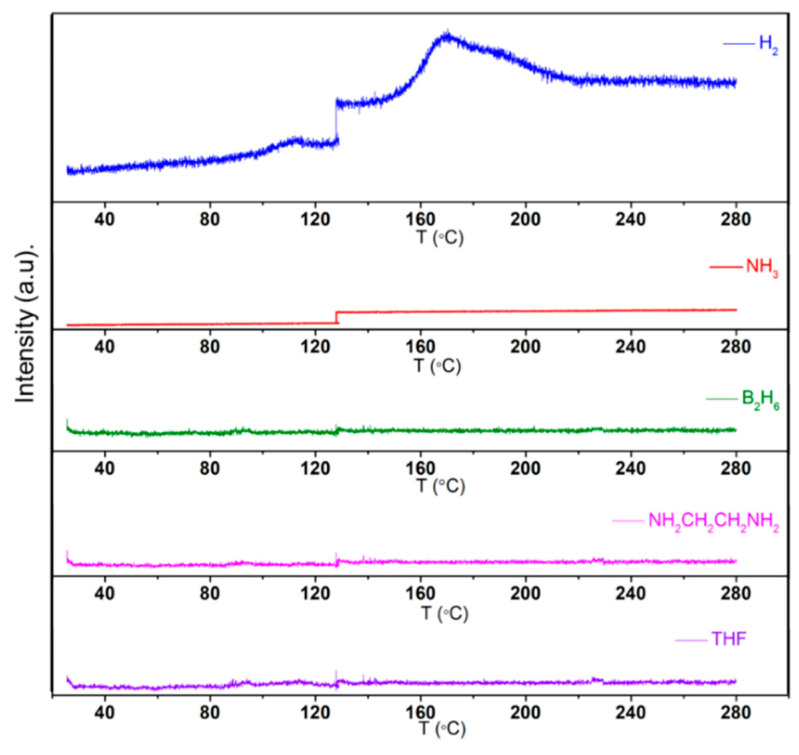
Mass spectrometry data for Li[Al(BH_3_NHCH_2_CH_2_NHBH_3_)_2_] decomposition.

The purity of the gas released during the thermal dehydrogenation of Li[Al(BH_3_NHCH_2_CH_2_NHBH_3_)_2_] was analyzed using temperature-programmed mass spectrometry (TPMS) in the temperature range from room temperature to 280 °C. The TPMS analysis was performed within a glovebox under an argon atmosphere to prevent contamination by moisture, as Li[Al(BH_3_NHCH_2_CH_2_NHBH_3_)_2_] is highly sensitive to it. Due to the small size of the crucible, the signals’ intensities for the released gases in the mass spectrometry results were not as high as that of Na(THF)[Al(BH_3_NHCH_2_CH_2_NHBH_3_)_2_]. Nevertheless, the hydrogen signals were visible, despite the challenges associated with detecting hydrogen due to its light weight. No signals corresponding to other gases were observed, confirming that ammonia (NH_3_), diborane (B_2_H_6_), ethylenediamine (NH_2_CH_2_CH_2_NH_2_), and THF were not released during the decomposition process. Only hydrogen was detected (Figure 9).

These findings indicate that Li[Al(BH_3_NHCH_2_CH_2_NHBH_3_)_2_] releases approximately 6.6 wt.% of pure hydrogen when heated to 280 °C. The successful formation of this novel Al-based amidoborane compound suggests that it effectively suppresses the release of unwanted by-products during thermal hydrogen desorption. These by-products are particularly prominent in NH_3_BH_3_ and are also observed, though to a lesser extent, in other metallic amidoboranes [12], such as NaNH_2_BH_3_, NaLi(NH_2_BH_3_)_2_, NaMg(NH_2_BH_3_)_3_, etc.

#### 2.3.2. Thermal Dehydrogenation of Na(THF)[Al(BH_3_NHCH_2_CH_2_NHBH_3_)_2_]

**Figure 10:**
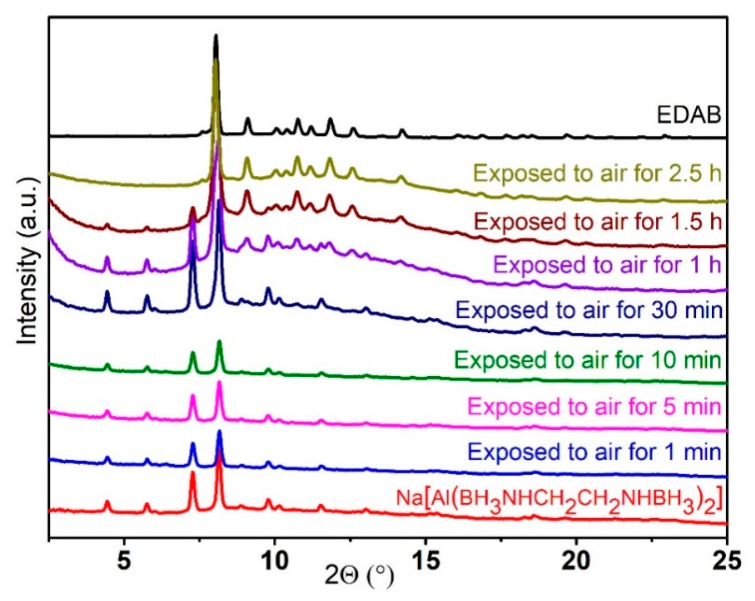
The PXRD patterns of Na(THF)[Al(BH_3_NHCH_2_CH_2_NHBH_3_)_2_], exposed to air for different periods of time, and the pattern of EDAB (λ = 0.71073 Å).

First, the stability of Na(THF)[Al(BH_3_NHCH_2_CH_2_NHBH_3_)_2_] in the air was investigated based on PXRD and compared to Li[Al(BH_3_NHCH_2_CH_2_NHBH_3_)_2_]. The Na-based compound demonstrated higher stability in ambient air (Figure 10). No changes were observed in the PXRD pattern of Na(THF)[Al(BH_3_NHCH_2_CH_2_NHBH_3_)_2_] during the initial 30 min of exposure to air. However, after 1 h, the peaks corresponding to EDAB appeared due to hydrolysis, and after 2.5 h of exposure, the PXRD pattern of Na(THF)[Al(BH_3_NHCH_2_CH_2_NHBH_3_)_2_] completely changed into that of EDAB.

The coordination of THF to Na^+^ in Na(THF)[Al(BH_3_NHCH_2_CH_2_NHBH_3_)_2_] may contribute to the better binding of [BH_3_NHCH_2_CH_2_NHBH_3_]^2−^ to Al^3+^. This may hinder the interaction of water molecules with both Al and Na, leading to the increased stability of Na(THF)[Al(BH_3_NHCH_2_CH_2_NHBH_3_)_2_] over a longer period of time in ambient air.

**Figure 11:**
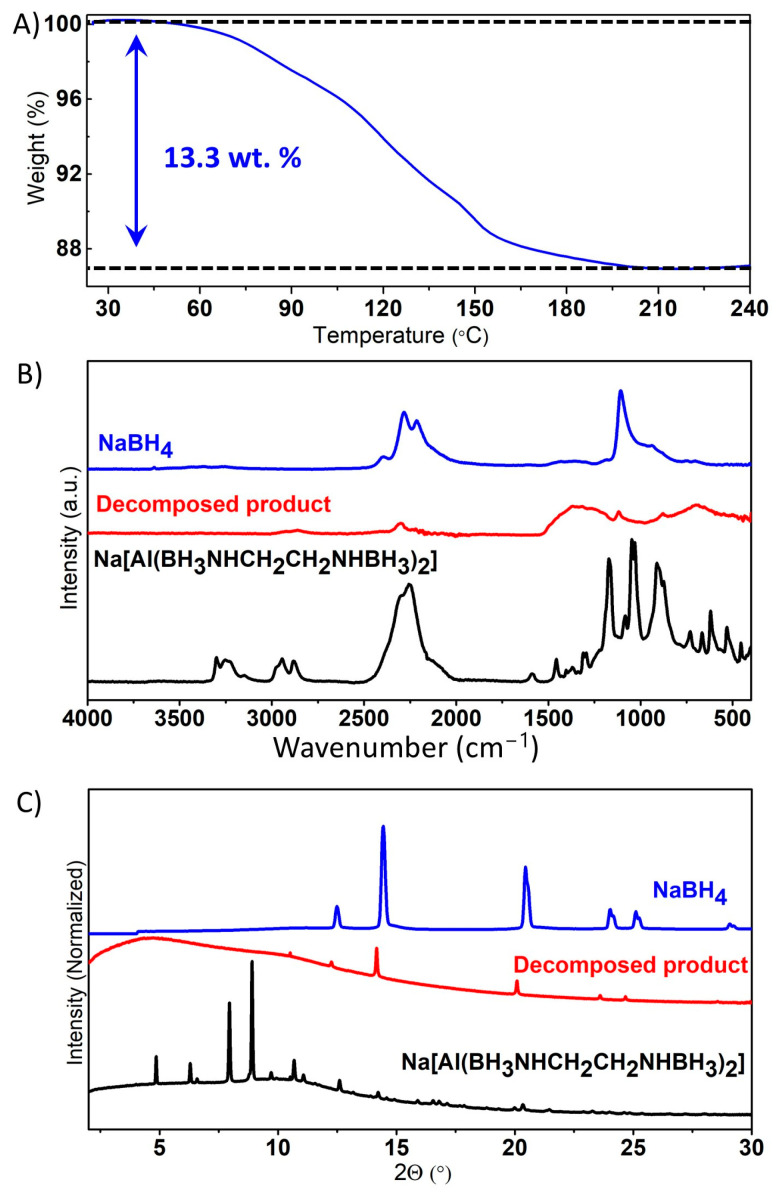
Thermogravimetric analysis (TGA) of Na(THF)[Al(BH_3_NHCH_2_CH_2_NHBH_3_)_2_] (**A**); ATR-IR spectra of Na(THF)[Al(BH_3_NHCH_2_CH_2_NHBH_3_)_2_], the thermally decomposed product containing NaBH_4_ (**B**); PXRD patterns of Na(THF)[Al(BH_3_NHCH_2_CH_2_NHBH_3_)_2_], the thermally decomposed product and of NaBH_4_ (**C**) (λ = 0.77509 Å).

Due to the good stability of Na(THF)[Al(BH_3_NHCH_2_CH_2_NHBH_3_)_2_] in the air, we studied its thermal decomposition under an inert nitrogen atmosphere using an instrument installed outside of a glovebox. The thermal decomposition of Na(THF)[Al(BH_3_NHCH_2_CH_2_NHBH_3_)_2_] occurs in a single step, initiated at approximately 55 °C and exhibiting a continuous weight loss without oscillation (jet effect), in contrast to the behavior observed in the case of EDAB [40] (Figure 11A). Compared to Li[Al(BH_3_NHCH_2_CH_2_NHBH_3_)_2_], decomposing at 104 °C, the sodium salt started to decompose at a lower temperature (55 °C). After heating to 240 °C, the solid decomposition products were identified as the known crystalline NaBH_4,_ along with some unknown crystalline and amorphous phases containing C-H bonds (Figure 11B,C). The formation of BH_4_^−^ during the thermal decomposition is similar to Na[Al(NH_2_BH_3_)_4_] [34] and Na[Al(CH_3_NHBH_3_)_4_] [37]. The mass loss during the thermal decomposition of Na(THF)[Al(BH_3_NHCH_2_CH_2_NHBH_3_)_2_] from room temperature to 240 °C was ~13.3 wt.%. This value exceeds the hydrogen content of Na(THF)[Al(BH_3_NHCH_2_CH_2_NHBH_3_)_2_], which is 5.4 wt.% (excluding H on carbon). This should be caused by the release of THF coordinated to Na^+^ evolving along with hydrogen during the thermal decomposition.

**Figure 12:**
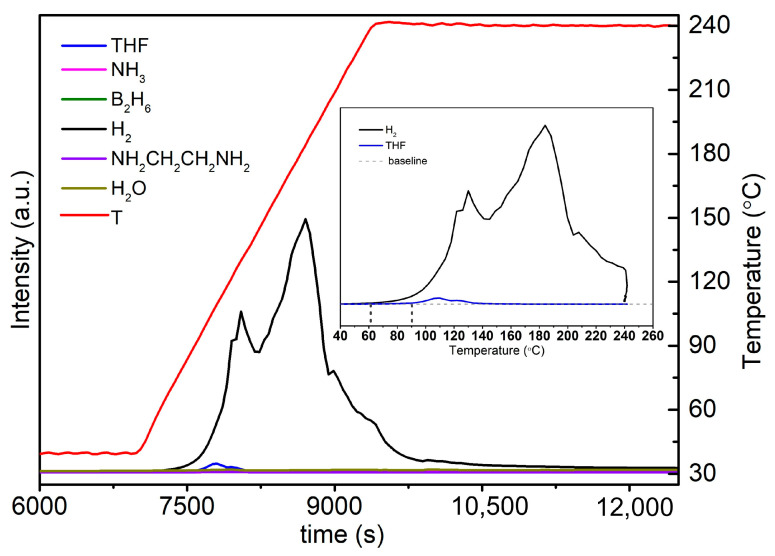
Mass spectrometry data for Na(THF)[Al(BH_3_NHCH_2_CH_2_NHBH_3_)_2_] decomposition.

To better understand the dehydrogenation of Na(THF)[Al(BH_3_NHCH_2_CH_2_NHBH_3_)_2_], we performed mass spectrometry analysis in the temperature range from 40 °C to 240 °C. The results revealed that hydrogen and THF were released. Notably, we did not find the signals of ammonia (NH_3_), diborane (B_2_H_6_), or ethylenediamine (NH_2_CH_2_CH_2_NH_2_), which are usually released along with H_2_ during the thermal dehydrogenation of NH_3_BH_3_ and its derivatives (Figure 12).

To avoid the influence of the THF on the hydrogen released from Na(THF)[Al(BH_3_NHCH_2_CH_2_NHBH_3_)_2_], we attempted to remove THF from Na(THF)[Al(BH_3_NHCH_2_CH_2_NHBH_3_)_2_] via pumping at elevated temperatures or via washing with other lower boiling point solvents (such as diethyl ether and dichloromethane). We also tried to synthesize Na[Al(BH_3_NHCH_2_CH_2_NHBH_3_)_2_] in other solvents such as toluene, 1,4-dioxane and dichloromethane, hexane, etc. However, all attempts were unsuccessful in removing THF from its adduct with Na[Al(BH_3_NHCH_2_CH_2_NHBH_3_)_2_] without the decomposition of the latter. From the mass spectrometry (Figure 12), we found that H_2_ is released from about 60 °C, and THF release started at about 90 °C. This is why we cannot remove THF via vacuum at elevated temperatures. Overall, the formation of Na(THF)[Al(BH_3_NHCH_2_CH_2_NHBH_3_)_2_] suppressed the liberation of common byproducts such as NH_3_ and B_2_H_6_.

## 3. Materials and Methods

All samples were obtained from commercially available NaAlH_4_ (93%), LiAlH_4_ (>98%), NaBH_4_ (97%), NH_2_CH_2_CH_2_NH_2_·2 HCl (98%), and anhydrous THF (≥99.9%), toluene (99.85%), hexane (≥99.9%), diethyl ether (≥99.5%), 1,4-dioxane (≥99.5%), and CH_2_Cl_2_ (99.5%), which were purchased from Sigma-Aldrich Co., Ltd., (Saint Louis, MO, USA). All operations were performed in a glovebox with a high-purity argon atmosphere.

### 3.1. Synthesis of BH_3_NH_2_CH_2_CH_2_NH_2_BH_3_ (EDAB)

**Figure 13:**
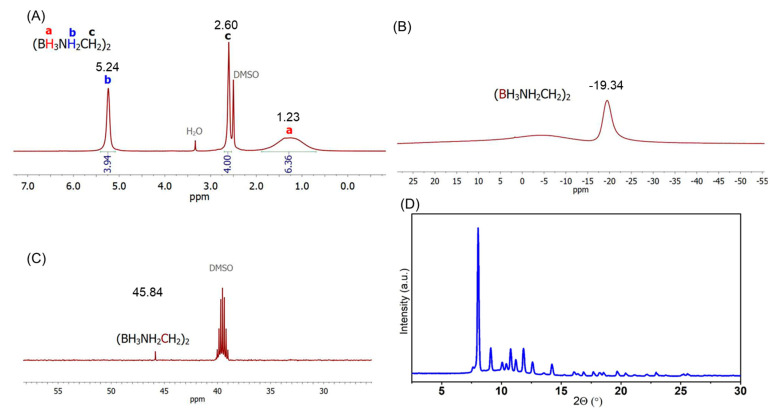
^1^H (**A**), ^11^B (**B**), ^13^C NMR (**C**) spectra and PXRD (**D**) pattern of EDAB (λ = 0.71073 Å).

EDAB was obtained through a procedure adapted from the literature [38]. In brief, the synthesis was performed as follows. Powdered NaBH_4_ (1.40 g, 37 mmol), NH_2_CH_2_CH_2_NH_2_·2 HCl (2.40 g, 18 mmol), and THF (250 mL) were added to a 500 mL, three-neck, round-bottom flask. The obtained suspension was then vigorously stirred at room temperature under an argon atmosphere for 48 h. Then, the solid co-product (NaCl) was removed from the reaction mixture by filtration, and the solvent of the collected filtrate was removed through evaporation under reduced pressure (using a rotary evaporator) to obtain EDAB. The white solid was washed with diethyl ether three times and then dried under a vacuum for 4 h to eliminate residual solvents. The product was characterized by means of ^1^H (Figure 13A), ^11^B (Figure 13B), and ^13^C (Figure 13C) NMR and PXRD (Figure 13D). Notably, the ^11^B NMR spectrum exhibits a broad peak instead of the expected multiplet. This phenomenon is likely attributed to the structural characteristics of EDAB. Due to its relatively long molecular framework, EDAB may undergo bending or folding, creating a more complex chemical environment for the terminal boron atoms. As a result, these boron nuclei are not only influenced by their directly bonded hydrogen atoms but also by additional intramolecular hydrogen interactions, which may disrupt the expected coupling patterns and lead to the observed broad peak.

### 3.2. Synthesis Method of Li[Al(BH_3_NHCH_2_CH_2_NHBH_3_)_2_]

**Figure 14:**
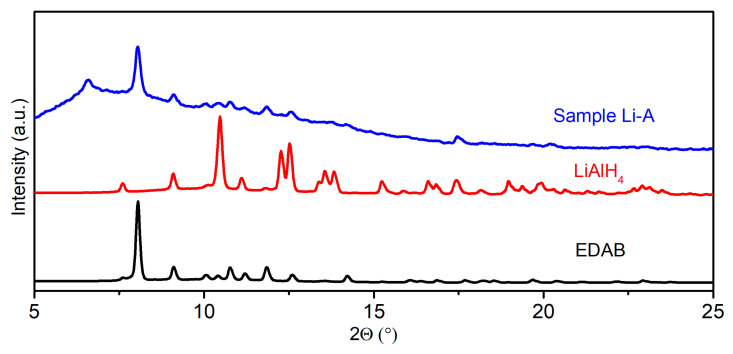
PXRD patterns of EDAB, LiAlH_4_, and Sample **Li-A** (λ = 0.71073 Å).

Sample **Li-A**: 1 eq. of LiAlH_4_ (26.9 mg, 0.7 mmol) and 2 eq. of EDAB (124.63 mg, 1.4 mmol) were placed into an 80 mL stainless steel vial with three 10 mm diameter stainless steel balls and milled in a planetary ball mill (Fritsch Pulverisette 7 Premium line, Fritsch, Markt Einersheim, Germany). The evolution of the gas pressure and temperature during the reaction was observed using the Easy GTM detection system accessory (Fritsch). The rotation speed was set to 600 rpm, and the ball-to-powder mass ratio was 30:1. The synthesis was performed using 240 milling cycles of 3 min milling interrupted by 5 min cooling breaks to yield dark gray powder. The product was characterized by means of PXRD (Figure 14).

Sample **Li-B**: The samples were produced using the same quantities of the reactants as for Sample **Li-A**. The rotation speed was set to 500 rpm, and the ball-to-powder mass ratio was 80:1. The synthesis was performed using 50 milling cycles of 30 min milling interrupted by 15 min cooling breaks to yield dark gray powder. The product was characterized by means of PXRD (Figure 1).

### 3.3. Synthesis Method of Na(THF)[Al(BH_3_NHCH_2_CH_2_NHBH_3_)_2_]

Powdered NaAlH_4_ (81 mg, 1.5 mmol), EDAB (263.3 mg, 3.0 mmol), and anhydrous THF (30 mL) were added to a 100 mL, one-neck, round-bottom Schlenk flask. The obtained suspension was then vigorously stirred at room temperature under an argon atmosphere for 24 (Sample **Na-A**), 48 (Sample **Na-B**), and 72 h (Sample **Na-C**), respectively. Then, the solvent was removed using a vacuum, and the resulting white solid was dried under a vacuum for another 5 h to eliminate residual solvents. The product was characterized by means of PXRD (Figure 3).

*Attempts for the Synthesis of Na[Al(BH_3_NHCH_2_CH_2_NHBH_3_)_2_] without THF solvents*

The procedure was similar to that of Sample **Na-B**. Powdered NaAlH_4_ (81 mg, 1.5 mmol), EDAB (263.3 mg, 3.0 mmol), and anhydrous toluene, hexane, diethyl ether, 1,4-dioxane, or CH_2_Cl_2_ (30 mL) were added to a 100 mL, one-neck, round-bottom Schlenk flask. The obtained suspension was then vigorously stirred at room temperature under an argon atmosphere for 48 h. Then, the solvent was removed using a vacuum, and the resulting white solid was dried under a vacuum for another 5 h to eliminate residual solvents.

*Attempts to remove THF solvents in Sample **Na-B***

A sample of **Na-B** powder (~100 mg) was placed into a glass tube and dried under a vacuum at 30 °C, 50 °C, and 70 °C for 48 h, using a tubular oven to remove THF solvents.

### 3.4. NMR Experiments

^1^H, ^13^C, and ^11^B nuclear magnetic resonance spectra were recorded on a Bruker Avance 500 MHz spectrometer (Bruker, Billerica, MA, USA). ^1^H, ^13^C, and ^11^B NMR chemical shifts are reported in ppm and calibrated against the residual signals of the deuterated DMSO.

### 3.5. Powder X-Ray Diffraction

Samples were carefully filled into 0.7 mm thin-walled glass capillaries (Hilgenberg GmbH, Malsfeld, Germany) within an argon-filled glovebox. To prevent contact with air, the capillaries were sealed with grease before being taken out of the glovebox. The sealed capillaries were then cut and promptly placed into wax on a goniometer head, ensuring that no air entered the capillary. Diffraction data were immediately collected using a MAR345 image-plate detector equipped with an Incoatec Mo (λ = 0.71073 Å) Microfocus (l µS 2.0) X-ray source operating at 50 kV and 1000 µA. The resulting two-dimensional images were azimuthally integrated using the Fit2D software (V17.006), with LaB_6_ serving as a calibrant.

Synchrotron PXRD patterns were collected with a PILATUS@SNBL diffractometer (SNBL, ESRF, Grenoble, France) equipped with a Dectris PILATUS 2M single-photon counting pixel area detector (λ = 0.77509 Å). Powder patterns were obtained by using raw data processed using the SNBL Toolbox software (https://soft.snbl.eu/snbltb/snbltb.html, version 2018.2.27, accessed on 20 February 2025), using the data for LaB_6_ as a standard.

### 3.6. Crystal Structure Determination

The synchrotron PXRD data for Na(THF)[Al(BH_3_NHCH_2_CH_2_NHBH_3_)_2_] were indexed in the tetragonal unit cell, and their structure was solved using global optimization using FOX software (version 1.9.7.0) [45]. The anions were modelled by conformationally free Z-matrices restraining bond distances and angles. Rietveld refinements were carried out in Fullprof [46], treating amidoboranes as separate rigid bodies within the complex anion and refining Na and Al as free atoms. The symmetry was confirmed using the ADDSYM routine in the program PLATON [47].

### 3.7. Fourier Transform Infrared Spectroscopy (FTIR)

Attenuated total reflectance (ATR)-IR spectra were recorded using a Bruker Alpha spectrometer. The spectrometer was equipped with a Platinum ATR sample holder, which featured a diamond crystal for single-bounce measurements. The entire experimental setup was located within an argon-filled glovebox to maintain an inert atmosphere during the measurements.

### 3.8. Thermogravimetric Analysis (TGA)

TGA measurements for the Li[Al(BH_3_NHCH_2_CH_2_NHBH_3_)_2_] sample were conducted using a Netzsch STA 449 F3 TGA/DSC (Netzsch, Selb, Germany). The TGA/DSC was equipped with a stainless-steel oven and located within an argon-filled glovebox to ensure an inert atmosphere during the measurements. The sample was loaded into Al_2_O_3_ crucibles and subjected to a heating rate of 5 °C/min under an argon flow of 100 mL/min.

TGA measurements for the Na(THF)[Al(BH_3_NHCH_2_CH_2_NHBH_3_)_2_] sample was conducted using a Mettler Toledo (New York, NY, USA) TGA/SDTA 851e instrument. The sample was placed in Al_2_O_3_ crucibles and subjected to a heating rate of 5 °C/min under a nitrogen flow of 100 mL/min.

### 3.9. Mass Spectrometry

Mass spectrometry analysis of Li[Al(BH_3_NHCH_2_CH_2_NHBH_3_)_2_] was carried out with the use of a Hiden Analytical HPR-20 QMS (Hiden Analytical Ltd., Warrington, UK) sampling system, which was installed in a glovebox. The samples were loaded into an Al_2_O_3_ crucible and heated from room temperature to 300 °C (5 °C/min) in an argon flow of 100 mL/min. Gas evolution was monitored by recording the highest intensity peak for each gas, i.e., m/z of 2, 17, 26, 30, and 42 for H_2_, NH_3_, B_2_H_6_, NH_2_CH_2_CH_2_NH_2_, and THF, respectively.

Mass spectrometry measurements for Na(THF)[Al(BH_3_NHCH_2_CH_2_NHBH_3_)_2_] were performed using a Hiden Catlab reactor combined with a quantitative gas analyzer (QGA) hidden quadrupole mass spectrometer (Hiden Analytical Ltd., Warrington, UK), which is installed outside of the glovebox. Before the experiment, samples were loaded into a quartz tube in between two layers of glass cotton under the protective atmosphere of an argon-filled glovebox. The two extremities of the quartz tube were sealed with Parafilm before being removed from the glovebox. The quartz tube was then installed in the sample holder outside the glovebox after quickly removing the Parafilm, and the argon flow (40 mL/min) was switched on immediately to prevent the contact of the sample with air. Samples were heated to 40 °C and kept isothermally for ~ 2 h to stabilize the temperature. Heating was then performed at a rate of 5 °C/min up to 240 °C corresponding to Na(THF)[Al(BH_3_NHCH_2_CH_2_NHBH_3_)_2_], followed by a 1 h isotherm. Gas evolution was monitored by recording the highest intensity peak for each gas, i.e., m/z of 2, 17, 18, 26, 28, 30, and 42 for H_2_, NH_3_, H_2_O, B_2_H_6_, N_2_, NH_2_CH_2_CH_2_NH_2_, and THF, respectively. H_2_O and N_2_ were monitored to check if the sample had come into contact with air or not.

## 4. Conclusions

We successfully synthesized Li[Al(BH_3_NHCH_2_CH_2_NHBH_3_)_2_] and Na(THF)[Al(BH_3_NHCH_2_CH_2_NHBH_3_)_2_] using mechanochemical synthesis and a wet chemical approach in THF from one equivalent of MAlH_4_ (M = Li and Na) and two equivalents of BH_3_NH_2_CH_2_CH_2_NH_2_BH_3_, respectively. In comparison to NH_3_BH_3_ and CH_3_NH_2_BH_3_, BH_3_NH_2_CH_2_CH_2_NH_2_BH_3_ exhibited higher inertness and required more energy input to form an Al-N bond when reacting with NaAlH_4_. This favors the reaction conditions between otherwise extremely reactive LiAlH_4_ and BH_3_NH_2_CH_2_CH_2_NH_2_BH_3_ through mechanochemical synthesis and thus avoids the explosive reaction that occurs between LiAlH_4_ and NH_3_BH_3_.

In the structure of Na(THF)[Al(BH_3_NHCH_2_CH_2_NHBH_3_)_2_], the Al^3+^ ion is tetrahedrally coordinated to two [BH_3_NHCH_2_CH_2_NHBH_3_]^2−^ anions through nitrogen atoms and forms chelates. The formation of the Al-N bond in Li[Al(BH_3_NHCH_2_CH_2_NHBH_3_)_2_] and Na(THF)[Al(BH_3_NHCH_2_CH_2_NHBH_3_)_2_] was confirmed through IR spectroscopy.

Thermogravimetric analysis (TGA) and mass spectrometry revealed that Li[Al(BH_3_NHCH_2_CH_2_NHBH_3_)_2_] releases 6.6 wt.% of pure hydrogen from room temperature to 280 °C. The TGA and mass spectrometry analysis of Na(THF)[Al(BH_3_NHCH_2_CH_2_NHBH_3_)_2_] indicated that the released hydrogen was contaminated with THF. Nevertheless, the formation of Li[Al(BH_3_NHCH_2_CH_2_NHBH_3_)_2_] and Na(THF)[Al(BH_3_NHCH_2_CH_2_NHBH_3_)_2_] effectively suppressed the release of by-products that typically contaminate hydrogen during the thermal dehydrogenation of NH_3_BH_3_ and its derivatives, such as NH_3_ and B_2_H_6_.

In this work, it has been confirmed that the substitution on the N-side of NH_3_BH_3_ allows the formation of stable chelate complexes that exhibit an effective suppression of the release of the common gaseous impurities. Furthermore, these compounds are the first examples of chelated aluminum amidoborane complexes.

## Figures and Tables

**Table 1:** Pressure and temperature at the start and at the end of synthesis, and the amount of released gas during synthesis of Sample **Li-B** using the Easy-GTM system.

P_start_ (bar)	T_start_ (°C)	P_end_ (bar)	T_end_ (°C)	Δn Gas (mmol)	Δn Gas/nLiAlH_4_
1.0	23.6	1.7	23.1	2.4	3.3

## Data Availability

CCDC number 2425724 contains supplementary crystallographic data for this paper. This data can be obtained free of charge from the Cambridge Crystallographic Data Center.
